# Longitudinal analysis shows GAA1 length and baseline clinical status as robust predictors of progression in Friedreich ataxia

**DOI:** 10.1007/s00415-026-13812-2

**Published:** 2026-04-09

**Authors:** Leire Manrique, Francisco Martínez-Dubarbie, Ana L. Pelayo-Negro, Natalia Benitez-Calle, María Victoria Sanchez-Pelaez, Daniel Cota-Gonzalez, Ruben Loza, Raquel Martinez-Díaz, Juan Irure-Ventura, Coro Sanchez-Quintana, Ivelisse Sanchez, Antoni Matilla-Dueñas, Jon Infante

**Affiliations:** 1 Neurology Department, Hospital Universitario de Navarra, Pamplona, Spain; 2 Neurology Department, Hospital Universitario Marqués de Valdecilla–IDIVAL, 39008 Santander, Spain; 3 Centro de Investigación Biomédica en Red Enfermedades Neurodegenerativas (CIBERNED), Madrid, Spain; 4 Department of Immunology, Hospital Universitario Marqués de Valdecilla-IDIVAL, Santander, Spain; 5 University of Cantabria (UC), Santander, Spain; 6 Neurogenetics Unit, Department of Neuroscience, Research Institute Germans Trias I Pujol (IGTP), Universitat Autònoma de Barcelona, Campus Can Ruti, Badalona, Barcelona, Spain; 7 Biointaxis S.L. Badalona, Barcelona, Spain

**Keywords:** Friedreich ataxia, Frataxin, Neurofilament light chain, Biomarkers, SARA, Disease progression

## Abstract

Friedreich’s ataxia (FRDA) is the most common early onset hereditary ataxia, caused by GAA repeat expansions in the *FXN* gene. The length of the shorter allele (GAA1) and age at onset are established determinants of disease severity, though additional biomarkers such as frataxin expression and neurofilament light chain (NfL) have been proposed. We conducted a 30-month prospective longitudinal study, including 25 FRDA patients and 16 heterozygous carriers, assessing clinical progression through SARA, FARS–ADL, INAS, EQ-5D, SCAFI, and CCFS scales. Baseline measures included GAA repeats, frataxin expression in fibroblasts, CSF NfL, and disease burden. Frataxin levels were significantly reduced in patients and correlated with GAA1 length and baseline severity. SARA, FARS–ADL, and INAS worsened significantly over time, while SCAFI and CCFS remained stable. GAA1 length and baseline SARA score emerged as the strongest predictors of progression. CSF NfL was elevated in younger patients and declined with age but did not correlate with severity or progression. These results support GAA1 length and baseline clinical status as robust predictors of progression and suggest limited utility of CSF NfL as a longitudinal biomarker particularly in later disease stages.

**Supplementary Information:**

The online version contains supplementary material available at 10.1007/s00415-026-13812-2.

## Introduction

Friedreich’s ataxia (FRDA) is the most common form of early onset hereditary ataxia. It is caused by biallelic GAA trinucleotide repeat expansions in the first intron of the *FXN* gene, located on chromosome 9 [1, 2]. In approximately 4–5% of patients, the genotype consists of compound heterozygosity, with a GAA expansion on one allele and a point mutation on the other [3–5]. GAA expansions suppress *FXN* gene transcription, leading to reduced tissue expression of frataxin—a mitochondrial protein essential for iron–sulphur cluster biogenesis—consequently resulting in mitochondrial dysfunction, neurodegeneration, and multisystem clinical manifestations [6]. The typical age at onset ranges between 10 and 15 years, with an average time to wheelchair dependence of 10–15 years after disease onset. However, both onset age and disease progression vary widely among individuals. FRDA usually begins in childhood with progressive gait and limb ataxia, accompanied by sensory neuropathy and dysarthria. Non-neurological manifestations are common and include skeletal deformities, diabetes mellitus, and cardiac involvement, such as hypertrophic cardiomyopathy or arrhythmias. Atypical forms have also been described, including late-onset FRDA (onset after 25 years) and very late-onset FRDA (onset after 40 years). In these cases, the phenotype more closely resembles a spastic ataxia with pyramidal signs, slower progression, and infrequent non-neurological complications [7]. To date, there is no cure for FRDA; however, omaveloxolone has recently become the first approved disease-modifying therapy [8, 9]. Several additional therapeutic approaches are under active investigation, mainly targeting frataxin upregulation, gene therapy, and mRNA or protein replacement strategies [10, 11]. Among the known prognostic variables, an earlier age at onset and a higher number of repeats in GAA1 are most strongly associated with more severe phenotypes and faster progression [12]. GAA1 length is inversely correlated with age at onset, indicating that larger expansions cause stronger gene silencing and lower frataxin levels, leading to more pronounced mitochondrial dysfunction and earlier disease manifestation [13]. However, these parameters alone do not fully account for the clinical heterogeneity observed among patients, suggesting the contribution of additional modifying factors. Frataxin levels measured in peripheral tissues (blood, buccal cells, fibroblasts) have been proposed as a direct functional biomarker of *FXN* gene expression. Several studies have shown that frataxin levels are significantly reduced in FRDA patients compared with controls, remain relatively stable over time, and correlate with the genotype and clinical severity [14, 15]. Nevertheless, available studies remain limited, often involve small cohorts, and few have systematically examined longitudinal changes or their relationship with clinical progression. Neurofilament light chain (NfL) has also emerged as a potential biomarker in FRDA, reflecting neuroaxonal injury and neurodegeneration. Elevated blood NfL levels have been reported in younger FRDA patients and in those with early disease onset compared with controls [16]. These levels remain relatively stable during follow-up and tend to decline with age, converging toward control values after approximately 40 years [16–18]. Despite these advances, important gaps remain. The relationship between tissue frataxin expression and clinical severity or progression, the impact of composite variables, such as disease burden (defined as GAA1 × disease duration) and the potential role of CSF NfL—yet to be studied in FRDA—are poorly understood. As novel therapeutic strategies and clinical trials are rapidly emerging, a more comprehensive understanding of the factors influencing disease severity and progression is crucial for optimizing patient selection, refining prognostic assessment, and identifying reliable biomarkers and clinical endpoints.

## Objectives

The aim of this study was to evaluate the association between disease severity and clinical progression—measured by changes in SARA, INAS, FARS–ADL, SCAFI, and CCFS scales—and biological biomarkers, including GAA1 repeat length, frataxin expression in fibroblasts, CSF NfL levels, and composite variables, such as disease burden. Through longitudinal follow-up, we sought to determine their potential value as predictors of disease trajectory and as candidate outcome measures for therapeutic trials.

## Methods

### Subjects and recruitment

Patients with genetically confirmed FRDA and asymptomatic heterozygous carriers were recruited through the Spanish Ataxia Association (FEDAES) and from our Center’s outpatient clinic at the University Hospital Marqués de Valdecilla in Santander, a referral center in Spain for the study of patients with degenerative ataxia. Inclusion criteria required a diagnosis of FRDA confirmed by genetic testing. Recruitment prioritized patients within 10 years of disease onset who remained ambulatory, although individuals with more advanced disease were also included. Exclusion criteria included participation in other clinical trials. Heterozygous carriers were identified among siblings or parents of affected participants. All participants provided written informed consent at baseline. The study was approved by the Institutional Ethics Committee of Instituto de Investigación Marqués de Valdecilla-IDIVAL in compliance with the ethical standards laid down in the 1964 Declaration of Helsinki and its later amendments.

### Clinical assessments and procedures

We conducted a prospective longitudinal study assessing clinical severity, functional scales, and biological biomarkers. Participants underwent four visits: the first three visits at 9-month intervals and a final visit at 12 months. Baseline data included demographic and clinical characteristics (age, sex, age at onset, medication, ambulation status, scoliosis, diabetes, and cardiomyopathy). Disease burden was calculated as the product of GAA1 repeat length and disease duration (in years).

### Clinical scales

At each visit, disease severity and function were assessed using standardized scales: SARA (Scale for the Assessment and Rating of Ataxia): evaluates ataxia severity across eight items (gait, stance, sitting, speech, finger chase, nose–finger test, alternating hand movements, heel–shin slide). Total scores range from 0 to 40, with higher scores indicating greater severity. FARS–ADL (Friedreich Ataxia Rating Scale–Activities of Daily Living): assesses disability across nine daily activities (walking, dressing, feeding, speech, writing, bladder control, and posture maintenance). Scores range from 0 to 36, with higher values reflecting greater disability. INAS (Inventory of Non-Ataxia Signs): a checklist of 16 non-ataxia neurological signs, scored as present or absent; total score (0–16) reflects extracerebellar involvement. EQ-5D-3L: administered by structured interview to assess health-related quality of life. SCAFI (Spinocerebellar Ataxia Functional Index): a composite measure combining three timed tests—8-m walk (8 MW), nine-hole peg test (9HPT, both hands), and the PATA repetition test for speech. Each subtest was converted into a Z-score and averaged to obtain a composite SCAFI score. For patients unable to perform a subtest due to severe ataxia, maximum values were assigned (8 MW =  1800 s; 9HPT = 3000 s; PATA = 0). CCFS (Composite Cerebellar Functional Severity Score): assesses upper-limb coordination and precision using two timed tasks (click test and 9HPT), combined using a standardized formula to yield a single score.

### Biological samples

Lumbar punctures were performed at baseline and at the third visit to measure cerebrospinal fluid (CSF) neurofilament light chain (NfL) levels, expressed in pg/mL and quantified by immunoassay (Lumipulse G). Skin biopsies were processed and fibroblasts obtained as previously reported [19]. To determine frataxin expression in fibroblasts, pellets were lysed in Tris–HCl (10 mM, pH 7.4) buffer containing 2% SDS, 15% glycerol, 50 mM sodium fluoride, 5 mM othovanadate, 1 mM EDTA, 1X Complete™ EDTA-free protease inhibitor cocktail (Roche Diagnostics). Twenty µgs of total protein per sample were separated on 15% polyacrylamide SDS–PAGE gels and transferred to nitrocellulose 0.45 µm pore membranes (PerkinElmer). After blocking with 0.5% casein in TBS (Thermo Fisher Scientific), membranes were incubated with anti-frataxin mouse monoclonal (Abcam; Ab113691, 1:5,000) overnight at 4 °C, and anti-GAPDH (Sigma-Aldrich,St. Louis, MO; G9545, 1:20,000) for 1 h. Infrared-dye conjugated secondary antibodies IRDye 800CW Donkey anti-Mouse IgG and IRDye 680RD and Donkey anti‐Rabbit IgG (LI-COR Biosciences) were used at 1:20,000. Immunoreactivity was analysed in the Odyssey Infrared Imaging System using Image Studio Lite software (LI-COR Biosciences). Frataxin levels in heterozygous carriers and patients were expressed as percentage relative to control values from non-carrier individuals.

GAA repeat lengths in both FXN alleles were determined by PCR followed by triple-primed PCR amplification of the expanded (GAA) n repeat in the intron 1 of the *FXN* gene and confirmation of expanded repeat length by long-range PCR and TP-PCR, as described [20].

### Outcomes

The primary outcome was the association between longitudinal changes in clinical scales (SARA, INAS, FARS–ADL, CCFS, SCAFI and EQ-5D) and biological biomarkers (GAA1 repeat length, CSF NfL concentration, and frataxin expression in fibroblasts).

### Statistical analysis

Normality was tested using the Shapiro–Wilk test. Data were summarized as mean ± SD or median (IQR), as appropriate. Non-normally distributed variables were log10-transformed. Correlations between continuous variables (e.g., FXN expression, age at onset, clinical scales) were analyzed using Pearson’s correlation. Group comparisons (patients vs. heterozygous carriers) were performed using Student’s *t* test. Repeated-measures ANOVA was used to compare longitudinal changes across visits, followed by Tukey’s post hoc tests when significant. Effect sizes were expressed as Cohen’s d. All comparisons were adjusted for age and sex.

Longitudinal changes were further examined using linear mixed-effects models (LMMs) with time (in days) as the independent variable and clinical scale scores as dependent variables. Covariates included age at onset, FXN expression, GAA1 repeat length, disease burden, disease duration, and their interactions with time. A base model (time as fixed effect; subject as random intercept) was compared against extended models using the corrected Akaike Information Criterion (AICc); the model with the lowest AICc was selected. Models within ΔAICc < 2 were considered equivalent.

Analyses were performed in RStudio (v4.4.0, R Foundation for Statistical Computing, Vienna, Austria). Missing data were handled by listwise deletion. A *p* value < 0.05 was considered statistically significant.

## Results

### Sample characteristics

A total of 41 participants were enrolled: 25 FRDA patients and 16 heterozygous carriers (60.9% women). The median age was 44.5 years (IQR 29–54.7), and the median follow-up duration was 940 days (IQR 917–998). Among FRDA patients, the median age was 31 years (IQR 19.5–49), and the median age at onset was 17 years (IQR 12–37.5). Summary data for demographics, CSF NfL, and frataxin expression are presented in Table 1.

**Table 1:** Baseline population characteristics

	Patients	Carriers	*p*
*n* (%)	25 (60.9%)	16 (39.1%)	< 0.05
Women, *n*. (%)	15 (60%)	10 (62.5%)	ns
Age (years), median (IQR)	17 (12–37.5)	55.5 (IQR 49.4–59.75)	< 0.05
Ambulatory, *n* (%)	16 (64%)	16 (100)	< 0.05
Short allele repeats GAA1 (*FXN* gene), median (IQR), *n*	682 (350–850)	6 (4–16)	< 0.05
Long allele repeats GAA2 (*FXN* gene), median (IQR), *n*	882 (850–1015)	915 (548.5–1115)	ns
Disease burden, median (IQR)	8235 (5665–14322)	−	
FXN levels in fibroblasts First visit, median (IQR)	0.3 (0.2–0.42)	0.51 (0.43–0.59)	< 0.05
NfL levels in CSF (pg/ml) First visit, median (IQR) Thrid visit, median (IQR)	453.5 (319–639) 460 (310–553)	351 (255–506) 347 (242–534)	< 0.05 < 0.05
SARA score	17.5 (11–25)	0 (0)	< 0.001
FARS–ADL score	10 (5.5–18)	0 (0)	< 0.001
INAS score	4 (2.5–5)	0 (0)	< 0.001
SCAFI composite Z score	−1.24 (−1.64–0.47)	0.68 (0–43-1)	< 0.001
CCFS Z score	1.3 (1.21–1.44)	0.9 (0.90–0.93)	< 0.001
EQ5D score	60 (40–77.5)	90 (80–90)	< 0.001

### Baseline findings

**Fig. 1:**
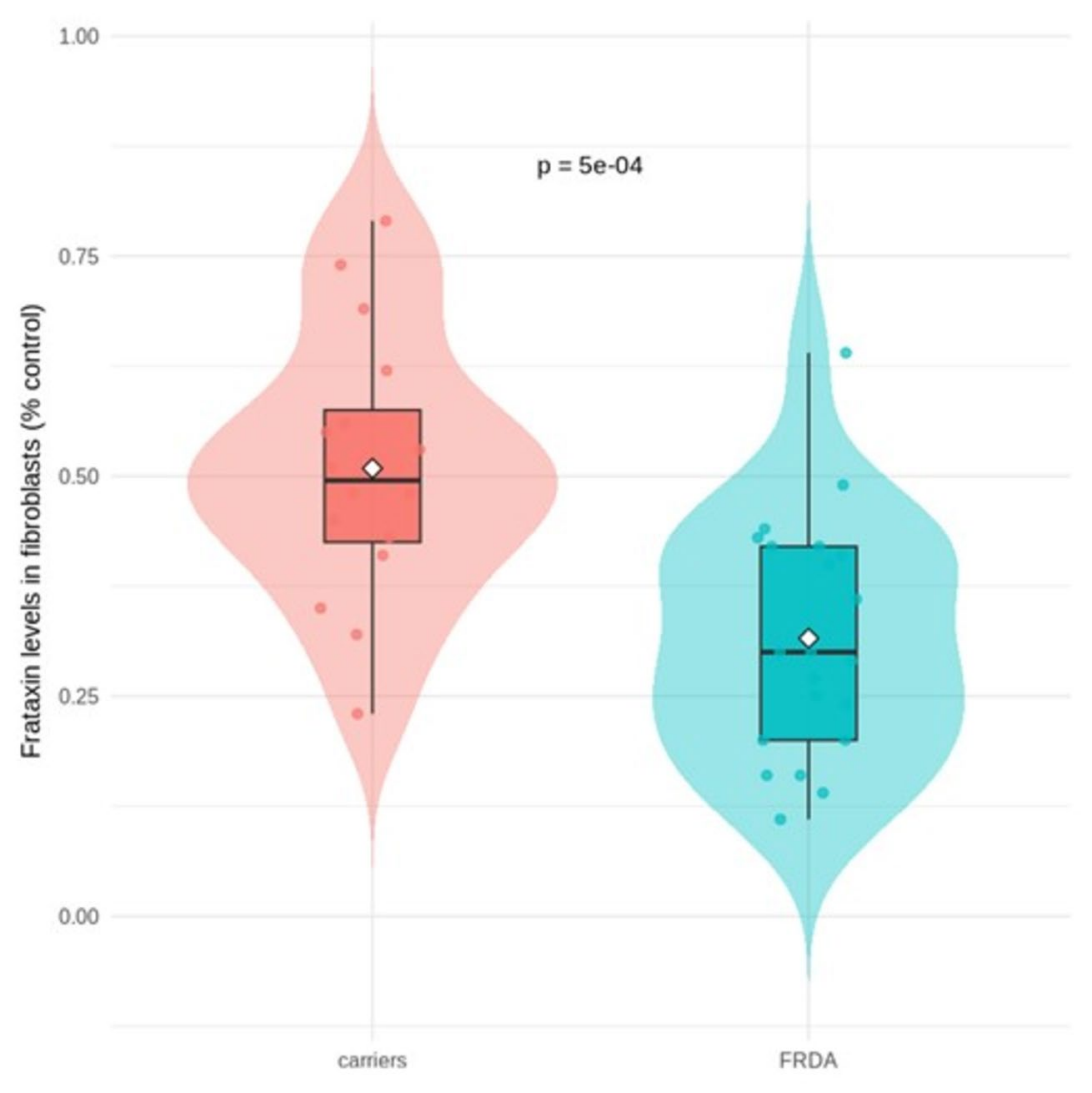
Frataxin expression levels in skin fibroblasts in FRDA patients and carriers at baseline. Values expressed relative to non-carrier individual laboratory controls

**Fig. 2:**
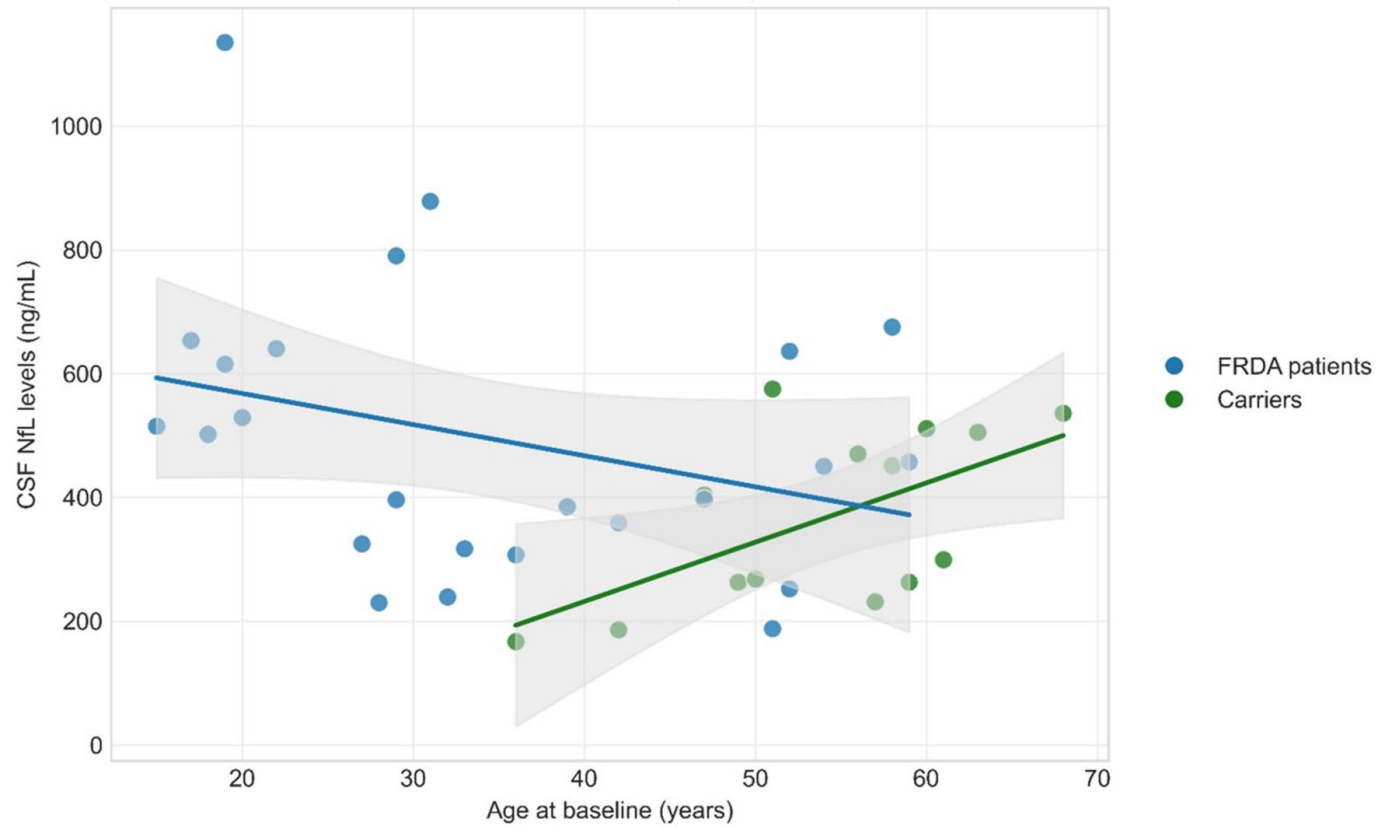
Baseline CSF NfL concentrations according to age in FRDA patients and carriers

FXN expression in fibroblasts was significantly lower in patients than in heterozygous carriers (difference = 0.36; 95% CI 0.31–0.40; *p* < 0.001; Cohen’s d = 1.28), although partial overlap was observed (Fig. 1). Baseline CSF NfL concentrations were higher in patients than in heterozygous carriers (*β* = 805.79; *p* = 0.01), even after adjusting for age and sex (Table 1). NfL dynamics differed by group: heterozygous carriers showed a marginally positive age-related trend (*β* = 10.23 pg/mL per year; *p* = 0.055), while patients showed a significant negative association (*β* =  −13.62; *p* = 0.024) (Fig. 2). At baseline, FRDA patients showed significantly worse performance across all clinical scales compared with heterozygous carriers (Table 1).

### Correlation analyses

FXN expression correlated inversely with GAA1 length (*r* =  −0.60; *p* < 0.001) and showed a trend toward correlation with age at onset (*r* = 0.39; *p* = 0.052). FXN levels were significantly correlated with disease severity across multiple scales: SARA (*r* =  −0.54), FARS–ADL (*r* =  −0.51), INAS (*r* =  −0.56), EQ5D (*r* = 0.53), SCAFI (*r* = 0.43), and CCFS (*r* =  −0.57) (all *p* < 0.01). CSF NfL did not correlate significantly with FXN levels, GAA1 length, disease burden, or any clinical scale.

GAA1 length showed a strong and inverse correlation with age at onset (*r* = −0.69; *p* < 0.001) and positively correlated with FARS–ADL, SARA, INAS, and CCFS clinical scales (all *p* < 0.001). Disease burden correlated with SARA (*r* = 0.67; *p* = 0.001), FARS–ADL (*r* = 0.66; *p* = 0.001), and INAS (*r* = 0.65; *p* = 0.001), but not with EQ5D (*r* = 0.06; *p* = 0.78) (Figure S1 A–E).

### Longitudinal clinical changes and baseline predictors

**Fig. 3:**
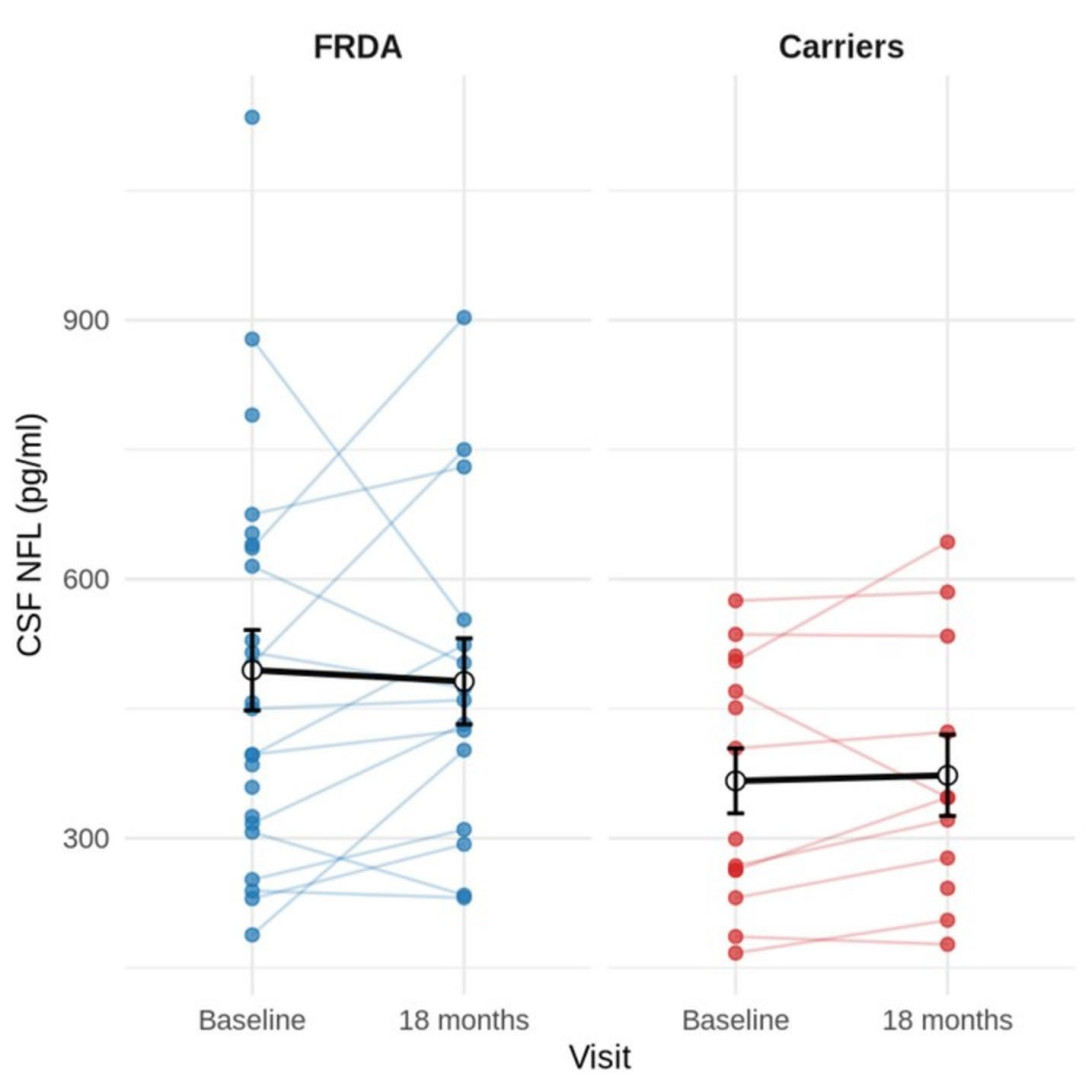
Longitudinal changes in CSF NfL across study visits in FRDA patients and carrier

Over the follow-up period, ambulation progressively declined: at baseline, 40% of patients were independently ambulant vs. 17% at study end. CSF NfL levels remained stable between baseline and the third visit (*p* = 0.056 overall; *p* = 0.21 among patients) (Fig. 3). SARA, FARS–ADL, and INAS scores worsened significantly (all *p* < 0.001), whereas EQ5D, SCAFI, and CCFS remained stable (Table 2).

**Table 2:** Clinical scale differences between timepoints in FRDA patients

Scale	Comparison	Mean difference	Adjusted *p* value	Cohen’s d
SARA	Baseline-9m	1.35	0.034	0.77
	Baseline-18m	2.03	< 0.001	1.19
	Baseline-30m	2.94	0.002	1.11
FARS-ADL	Baseline-9m	1.41	0.56	−
	Baseline-18m	3.18	0.03	0.79
	Baseline-30m	4.41	0.01	0.86
INAS	Baseline-9m	0.35	0.66	−
	Baseline-18m	1.12	0.01	0.92
	Baseline-30m	1.24	0.04	0.74
SCAFI	Baseline-9m	0.09	0.99	−
	Baseline-18m	0.13	0.99	−
	Baseline-30m	0.11	0.99	−
CCFS	Baseline-9m	0.01	0.99	−
	Baseline-18m	0.05	0.38	−
	Baseline-30m	0.03	0.99	−
EQ5D	Baseline-9m	0.71	0.99	−
	Baseline-18m	0.01	0.99	−
	Baseline-30m	1.65	0.99	−

The mean annual progression rate of SARA was approximately 1.0 point/year (SE = 0.19; *p* < 0.0001). Progression was faster in ambulatory patients (1.14 points/year; *p* < 0.001) than in non-ambulatory ones (0.76 points/year; *p* = 0.07).

Linear mixed-effects modelling identified baseline GAA1 repeat length and disease burden as significant predictors of the FARS–ADL slope (*p* = 0.02 and *p* = 0.04, respectively). For SARA, progression was significantly associated with GAA1 length (p < 0.001), baseline SARA score (*p* < 0.001), and disease duration (*p* = 0.02). FXN expression was associated with lower baseline SARA scores (*p* = 0.02). For INAS, age at onset (*p* < 0.001) and disease duration (*p* = 0.007) influenced baseline scores. CSF NfL showed no association with either baseline or longitudinal measures of any clinical scale.

## Discussion

In this study, we examined molecular, biological, and clinical markers reflecting disease severity and progression in FRDA. Our findings indicate that frataxin deficiency measured in fibroblasts, disease duration, and GAA1 repeat length are the strongest predictors of baseline disease severity. However, only GAA1 length and baseline SARA score predicted longitudinal decline, while CSF NfL showed limited utility as a biomarker of disease progression. Over time, SARA, FARS–ADL, and INAS scores worsened significantly during the study period, whereas EQ-5D, SCAFI, and CCFS remained stable. The mean annual progression on the SARA scale was approximately 1 point per year, differing between non-ambulatory (0.76 points/year) and ambulatory patients (1.14 points/year). Although both groups showed significant longitudinal changes, non-ambulatory patients exhibited a slower rate of progression, likely due to the ceiling effect of the SARA scale. Nevertheless, consistent with previous natural history studies, SARA remains a robust and sensitive measure of clinical progression in FRDA [13, 21, 22]. Our annual SARA progression rates are comparable to those reported in the EFACTS cohort—the largest longitudinal FRDA cohort to date—where the average yearly increase was 0.82 points, higher in ambulatory (1.12) than non-ambulatory (0.5) patients. Similarly, this study found an annual FARS–ADL progression rate of 1.3 points, with no significant differences between ambulatory and non-ambulatory subjects. Since both scales sensitively capture disease progression, FARS–ADL might be more suitable for advanced, non-ambulatory stages, while SARA appears more appropriate for earlier disease stages and shorter follow-up intervals (e.g., 9 months), as supported by our findings [21]. In contrast, composite functional measures (SCAFI and CCFS), designed to complement SARA due to its ceiling effect, showed no significant changes over the follow-up, indicating limited sensitivity to detect progression in small cohorts or over short periods of time. Similarly, EQ-5D, a generic quality-of-life scale, remained unchanged. In other longitudinal studies, these measures have shown some responsiveness, albeit less than SARA, mFARS, or FARS–ADL [13, 21]. It is likely that our limited sample size prevented detection of smaller changes on these scales.

Among baseline variables, only SARA score and GAA1 length were associated with faster progression on both the SARA and FARS–ADL scales, highlighting the predictive value of baseline disease status. This aligns with EFACTS cohort data, where subjects with lower baseline SARA scores exhibited faster annual progression [21, 23]. In this study, patients with longer GAA1 repeats had earlier disease onset, more severe baseline phenotypes, and faster deterioration on SARA and FARS–ADL scales, findings consistent with the FACOMS cohort and other studies [13, 24]. Unlike GAA1 length, age at onset did not predict faster progression, although it correlated with greater baseline severity. This discrepancy with FACOMS findings may stem from differences in the ataxia severity scales used (mFARS in FACOMS vs. SARA in EFACTS and this study) [12, 13, 24]. Disease burden, a variable dependent on GAA1 length, also predicted severity and progression on FARS–ADL.

Reduced frataxin expression is considered the primary driver of FRDA pathophysiology. Previous studies have demonstrated reduced frataxin levels in various tissues, approximately 20–40% of control levels in patients and 40–60% in heterozygous carriers [14, 15]. However, whether the extent of frataxin reduction influences clinical progression has not been systematically assessed in longitudinal cohorts. In this study, fibroblast frataxin expression was significantly lower in patients than in heterozygous carriers, although some overlap existed. Similar overlap between heterozygous carriers and late-onset FRDA cases has been reported in PBMCs, with intermediate levels observed in carriers [15]. These findings suggest that partial frataxin deficiency above a certain threshold may not be sufficient to produce the phenotype, implying additional contributing factors affecting frataxin functionality [25]. Frataxin levels in our cohort correlated with GAA1 length, as well as with multiple clinical (SARA, FARS–ADL, and INAS), functional (SCAFI, CCFS), and quality-of-life (EQ-5D) measures. However, absolute frataxin levels did not predict longitudinal change. In contrast, GAA1 length—strongly correlated with frataxin expression—was associated with both baseline severity and progression slopes. These results indicate that GAA1 length is a more reliable predictor of disease severity and progression than fibroblast frataxin expression. One explanation may be that peripheral frataxin expression does not accurately mirror changes in affected neural tissues, where genetic mosaicism or tissue-specific epigenetic regulation may play a role [26–28]. In summary, although peripheral frataxin expression correlates with both GAA1 length and disease severity, it does not improve upon GAA1 as a progression biomarker.

Neurofilament light chain (NfL) has emerged as a robust biomarker of disease severity and progression across neurodegenerative disorders, such as Alzheimer’s disease and Parkinson diseases among others, where elevated concentrations generally indicate increased neuroaxonal injury. In FRDA, however, NfL displays an atypical profile: levels are elevated relative to controls yet decline with advancing age and disease duration. To date, NfL in FRDA has been assessed exclusively in serum or plasma, leaving its precise anatomical origin unresolved. In this study, CSF NfL reproduced the paradoxical pattern described in peripheral blood. Although CSF NfL concentrations were higher in patients than in heterozygous carriers, their age-related trajectories differed markedly: heterozygous carriers showed the expected age-associated increase, whereas patients exhibited a significant age-associated decline, indicating a distinct age-dependent NfL behaviour in FRDA. CSF NfL was substantially elevated in younger patients at baseline and remained stable over 18 months, consistent with prior observations. [16–18, 29, 30] These data are consistent with the hypothesis that elevated serum NfL in FRDA may largely reflect degeneration of the dorsal root ganglia and sensory roots, which represent the earliest and most severely affected structures in the disease [31–33]. Given their anatomical proximity to the subarachnoid space, axonal injury in these regions could plausibly contribute to NfL release into cerebrospinal fluid and subsequently into the systemic circulation. However, direct evidence identifying the relative contribution of specific anatomical sources to circulating NfL levels in FRDA is currently lacking. Although the mechanistic basis of the NfL trajectory in FRDA remains uncertain, the described age-dependent pattern, with higher circulating NfL concentrations in younger individuals and lower levels in older patients suggests that, in FRDA, NfL may be more sensitive to phases of relatively greater neuroaxonal injury earlier in the disease course rather than reflecting cumulative disability in a linear fashion. One proposed explanation is that neuroaxonal loss may be more active during earlier stages, whereas in later phases fewer structurally intact axons remain available to release NfL into biofluids [18]. However, this interpretation remains hypothetical, and alternative mechanisms have also been suggested [16]. Further longitudinal and mechanistic studies are required to clarify the biological basis of this trajectory. In line with this uncertainty regarding the biological and clinical significance of NfL dynamics in FRDA, CSF NfL did not correlate with frataxin levels, GAA1 repeat length, disease burden, age at onset, or clinical measures, and longitudinal changes were not associated with clinical progression. Collectively, these results indicate that while CSF NfL may be a sensitive marker of early disease activity in FRDA, its limited dynamic range in later stages reduces its utility as a longitudinal biomarker.

Several limitations should be acknowledged. First, the cohort was intentionally designed to resemble populations typically enrolled in interventional clinical trials, prioritizing ambulant individuals with FRDA. This approach may introduce selection bias and limit the generalizability of our findings across the full clinical spectrum of the disease. Although the cohort included a range of disease severities and demographic characteristics, caution is warranted when extrapolating these results to more advanced or non-ambulant patients. In addition, all controls were heterozygous carriers and relatives of affected individuals, which may have influenced self-reported outcomes, such as EQ-5D. This should be considered when interpreting quality-of-life comparisons, although it is unlikely to affect the biological findings. Finally, heterozygous carriers were older than the patients; despite adjustment for age, this difference may still represent a potential limitation of the study.

In conclusion, this study shows that the SARA and FARS–ADL scales reliably detect clinical progression in FRDA, even in relatively small cohorts and over short observation periods. GAA1 repeat length and baseline SARA scores emerged as the strongest predictors of both disease severity and subsequent progression. Although frataxin expression in fibroblasts correlates closely with GAA1 length, it does not offer additional prognostic value. Finally, we show for the first time that CSF NfL dynamics in FRDA parallel those reported in serum, providing support for the hypothesis that the latter results from CSF diffusion; however, its distinctive age-related trajectory and lack of association with clinical severity or progression limit its usefulness as a longitudinal biomarker.

## Supplementary Information

Below is the link to the electronic supplementary material.

Supplementary file1 Correlation between CSF NfL baseline levels (A), FXN baseline levels (B), GAA1 (C), age at onset (D) and Disease Burden (E) with scales (DOCX 611 KB)

## Data Availability

Data supporting the findings of this study are available from the corresponding author upon reasonable request.

## Declarations

### AI use statement

No generative AI was used to create or edit the manuscript.

## References

[1] Harding AE (1981) Friedreich’s ataxia: a clinical and genetic study of 90 families with an analysis of early diagnostic criteria and intrafamilial clustering of clinical features. Brain 104:589–620 7272714 10.1093/brain/104.3.589

[2] Campuzano V, Montermini L, Moltó MD, Pianese L, Cossée M, Cavalcanti F et al (1996) Friedreich’s ataxia: autosomal recessive disease caused by an intronic GAA triplet repeat expansion. Science 271:1423–1427 8596916 10.1126/science.271.5254.1423

[3] Pandolfo M (2009) Friedreich ataxia: the clinical picture. J Neurol 256(Suppl 1):3–8 19283344 10.1007/s00415-009-1002-3

[4] Keita M, McIntyre K, Rodden LN, Schadt K, Lynch DR (2022) Friedreich ataxia: clinical features and new developments. Neurodegener Dis Manag 12:267–283 35766110 10.2217/nmt-2022-0011 PMC9517959

[5] Cossée M, Dürr A, Schmitt M, Dahl N, Trouillas P, Allinson P et al (1999) Friedreich’s ataxia: point mutations and clinical presentation of compound heterozygotes. Ann Neurol 45(2):200–206 9989622 10.1002/1531-8249(199902)45:2<200::aid-ana10>3.0.co;2-u

[6] Lynch DR, Farmer G (2021) Mitochondrial and metabolic dysfunction in Friedreich ataxia: update on pathophysiological relevance and clinical interventions. Neuronal Signal. 10.1042/NS20200093 34046211 10.1042/NS20200093 PMC8132591

[7] Bürk K (2017) Friedreich ataxia: current status and future prospects. Cerebellum Ataxias 4:4 28405347 10.1186/s40673-017-0062-x PMC5383992

[8] Lynch DR, Chin MP, Delatycki MB, Subramony SH, Corti M, Hoyle JC et al (2021) Safety and efficacy of omaveloxolone in Friedreich ataxia (MOXIe study). Ann Neurol 89(2):212–225 33068037 10.1002/ana.25934 PMC7894504

[9] Lynch DR, Chin MP, Boesch S, Delatycki MB, Giunti P, Goldsberry A et al (2022) Efficacy of omaveloxolone in Friedreich’s ataxia: delayed-start analysis of the MOXIe extension. Mov Disord 37:1703–1713 10.1002/mds.29286 36444905

[10] Scott V, Delatycki MB, Tai G, Corben LA (2024) New and emerging drug and gene therapies for Friedreich ataxia. CNS Drugs 38:791–805 39115603 10.1007/s40263-024-01113-z PMC11377510

[11] Zesiewicz TA, Hancock J, Ghanekar SD, Kuo SH, Dohse CA, Vega J (2020) Emerging therapies in Friedreich’s ataxia. Expert Rev Neurother 20:1215–1228 32909841 10.1080/14737175.2020.1821654 PMC8018609

[12] Rummey C, Corben LA, Delatycki M, Wilmot G, Subramony SH, Corti M et al (2022) Natural history of Friedreich ataxia. Neurology 99:e1499–e1510 35817567 10.1212/WNL.0000000000200913 PMC9576299

[13] Patel M, Isaacs CJ, Seyer L, Brigatti K, Gelbard S, Strawser C et al (2016) Progression of Friedreich ataxia. Ann Clin Transl Neurol 3:684–694 27648458 10.1002/acn3.332 PMC5018581

[14] Lazaropoulos M, Dong Y, Clark E, Greeley NR, Seyer LA, Brigatti KW et al (2015) Frataxin levels in peripheral tissue in Friedreich ataxia. Ann Clin Transl Neurol 2:831–842 26339677 10.1002/acn3.225 PMC4554444

[15] Saccà F, Puorro G, Antenora A, Marsili A, Denaro A, Piro R et al (2011) A combined nucleic acid and protein analysis in Friedreich ataxia. PLoS ONE 6:e17627 21412413 10.1371/journal.pone.0017627 PMC3055871

[16] Rummey C, Thomas-Black G, Garcia-Moreno H, Lynch DR, Abeti R, Arisoy H et al (2025) Increase of plasma biomarkers in Friedreich’s ataxia. Mov Disord 40:1863–1873 40498047 10.1002/mds.30250 PMC12485593

[17] Zeitlberger AM, Thomas-Black G, Garcia-Moreno H, Foiani M, Heslegrave AJ, Zetterberg H et al (2018) Plasma markers of neurodegeneration are raised in Friedreich’s ataxia. Front Cell Neurosci 12:366 30425621 10.3389/fncel.2018.00366 PMC6218876

[18] Hayer SN, Liepelt I, Barro C, Wilke C, Kuhle J, Martus P et al (2020) NfL and pNfH are increased in Friedreich’s ataxia. J Neurol 267:1420–1430 32002649 10.1007/s00415-020-09722-6 PMC7184046

[19] Corral-Juan M, Casquero P, Giraldo-Restrepo N et al (2022) New spinocerebellar ataxia subtype caused by SAMD9L mutation (SCA49). Brain Commun 4(2):fcac030 35310830 10.1093/braincomms/fcac030 PMC8928420

[20] Ciotti P, Di Maria E, Bellone E, Ajmar F, Mandich P (2004) Triplet repeat primed PCR (TP PCR) in molecular diagnostic testing for Friedreich ataxia. J Mol Diagn 6(4):285–289 15507666 10.1016/S1525-1578(10)60523-5 PMC1867489

[21] Reetz K, Dogan I, Hilgers RD et al (2021) Progression characteristics of EFACTS: a 4-year cohort study. Lancet Neurol 20:362–372 33770527 10.1016/S1474-4422(21)00027-2

[22] Reetz K, Dogan I, Hilgers RD et al (2016) Progression characteristics of EFACTS: a 2-year cohort study. Lancet Neurol 15:1346–1354 27839651 10.1016/S1474-4422(16)30287-3

[23] Porcu L, Fichera M, Nanetti L et al (2023) Longitudinal changes of SARA scale in Friedreich ataxia. Ann Clin Transl Neurol 10:2000–2012 37641437 10.1002/acn3.51886 PMC10647003

[24] Regner SR, Wilcox NS, Friedman LS et al (2012) Friedreich ataxia clinical outcome measures. J Child Neurol 27:1152–1158 22752494 10.1177/0883073812448462 PMC3674496

[25] Santoro M, Perna A, La Rosa P et al (2020) Compound heterozygosity for an expanded (GAA) and a (GAAGGA) repeat at FXN locus: from a diagnostic pitfall to potential clues to the pathogenesis of Friedreich ataxia. Neurogenetics 21(4):279–287 32638185 10.1007/s10048-020-00620-7

[26] Montermini L, Kish SJ, Jiralerspong S, Lamarche JB, Pandolfo M (1997) Somatic mosaicism in Friedreich’s ataxia. Neurology 49:606–610 9270608 10.1212/wnl.49.2.606

[27] Long A et al (2017) Somatic instability of the expanded GAA repeats. PLoS ONE 12:e0189990 29261783 10.1371/journal.pone.0189990 PMC5736210

[28] Evans-Galea MV, Carrodus N, Rowley SM et al (2012) FXN methylation predicts expression and clinical outcome. Ann Neurol 71:487–497 22522441 10.1002/ana.22671

[29] Clay A, Obrochta KM, Soon RK, Russell CB, Lynch DR (2020) Neurofilament light chain as biomarker. J Neurol 267:2594–2598 32385683 10.1007/s00415-020-09868-3

[30] Johnsson M, Zetterberg H, Blennow K, Lindberg C (2024) Plasma neurofilament in Friedreich ataxia. Heliyon 10:e24158 38163227 10.1016/j.heliyon.2023.e23347 PMC10755300

[31] Koeppen AH (2011) Friedreich’s ataxia: pathology and genetics. J Neurol Sci 303:1–12 21315377 10.1016/j.jns.2011.01.010 PMC3062632

[32] González-Cabo P, Palau F (2013) Mitochondrial pathophysiology in Friedreich’s ataxia. J Neurochem 126:53–64 23859341 10.1111/jnc.12303

[33] Stepanova A, Magrané J (2020) Mitochondrial dysfunction in neurons in Friedreich’s ataxia. Mol Cell Neurosci 102:103419 31770591 10.1016/j.mcn.2019.103419

