# Supplementary figures and images for: Longitudinal analysis shows GAA1 length and baseline clinical status as robust predictors of progression in Friedreich ataxia
Source: J Neurol. 2026 Apr 9;273(5):259. doi: 10.1007/s00415-026-13812-2 (PMC13065527; doi:10.1007/s00415-026-13812-2)

**Figure S1**


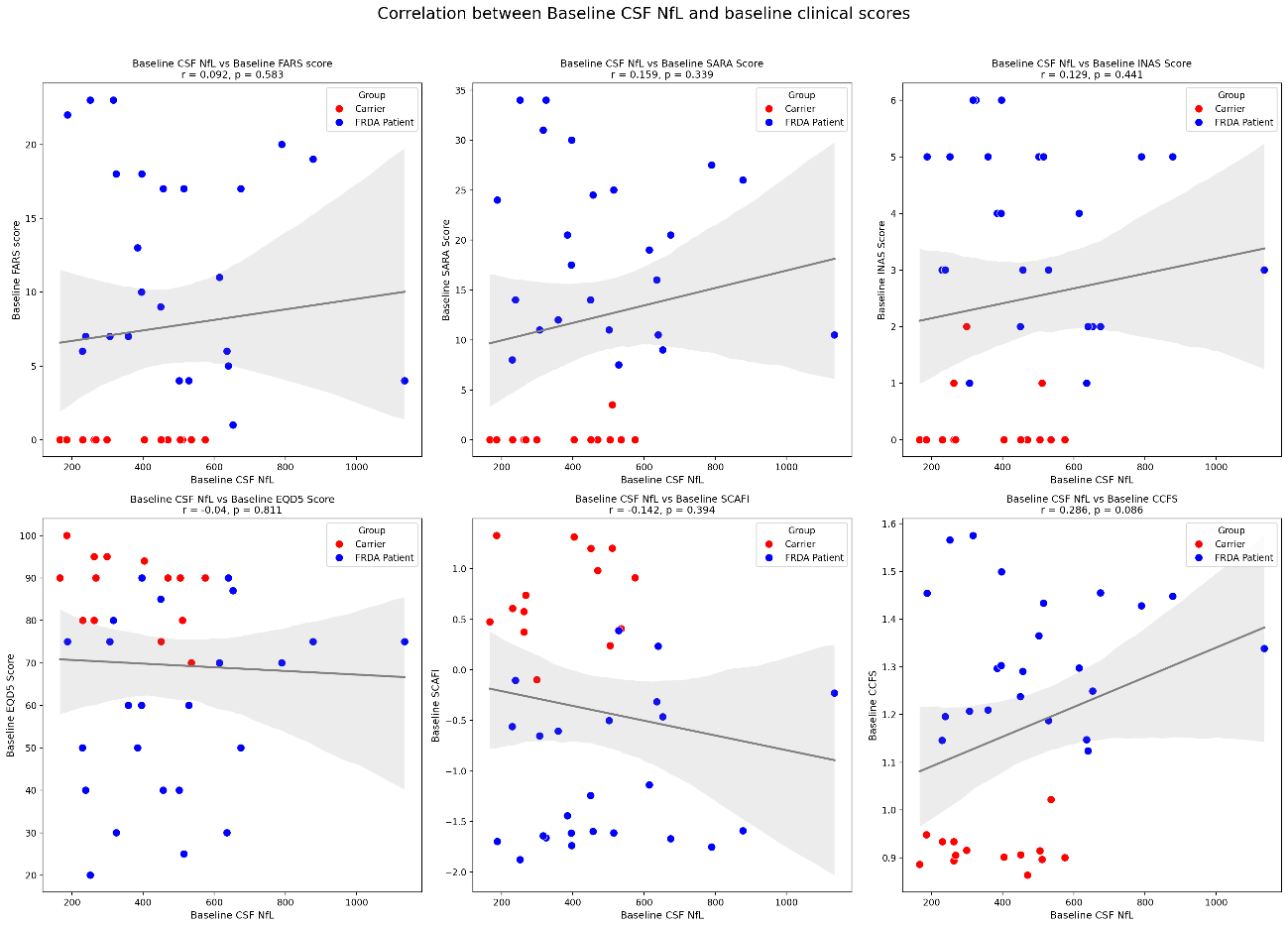


A


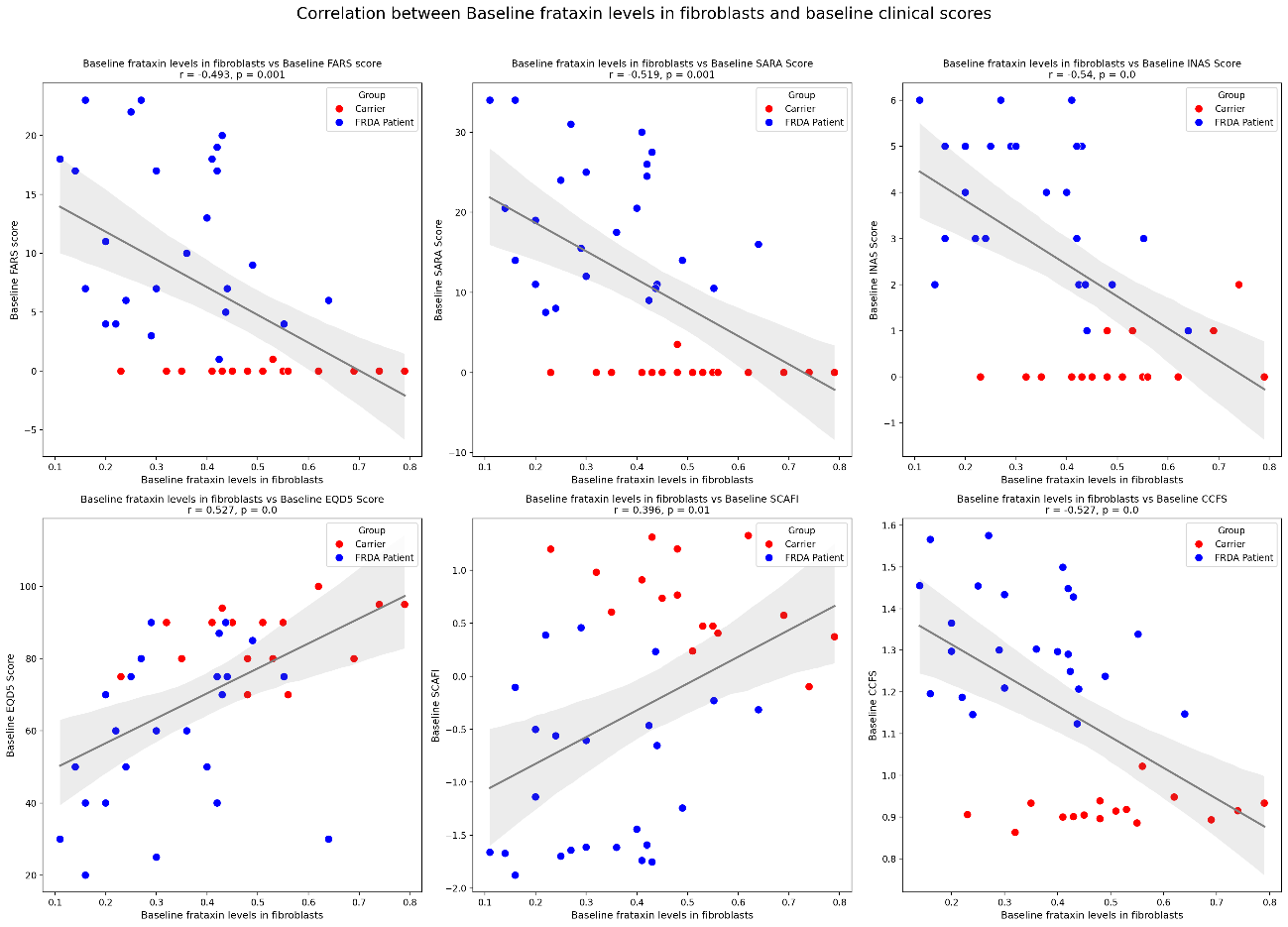


B


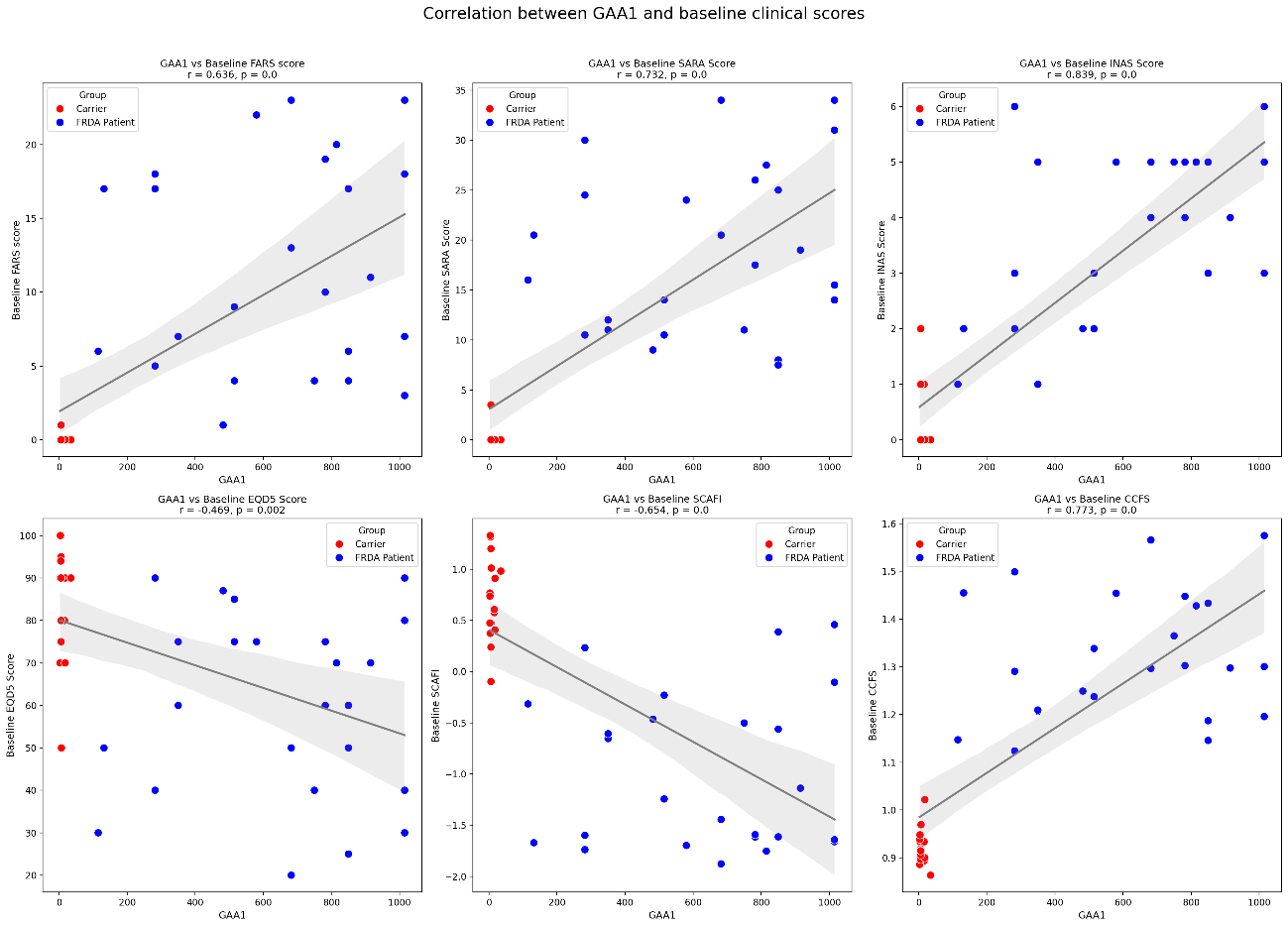


C


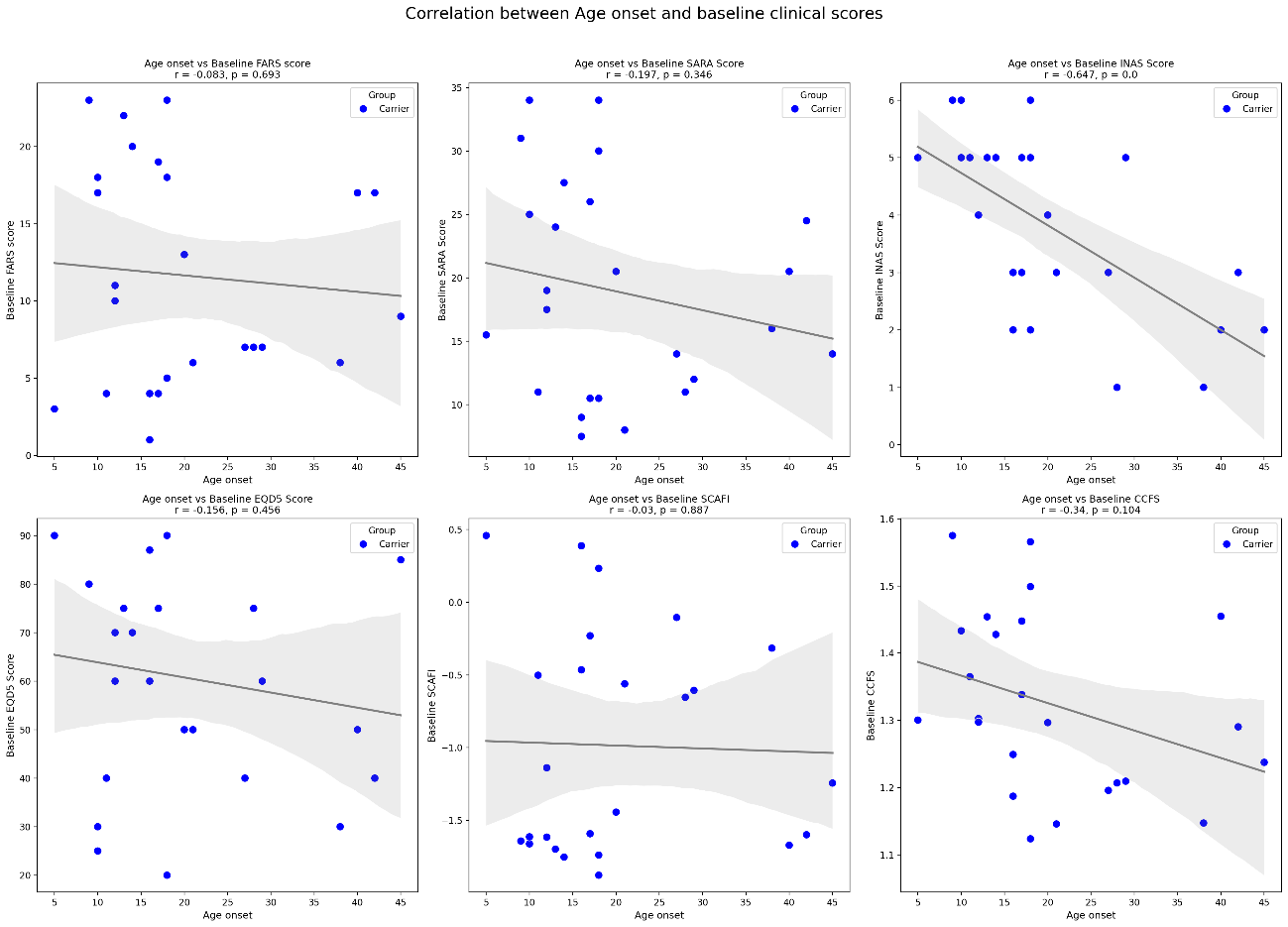


D

EE


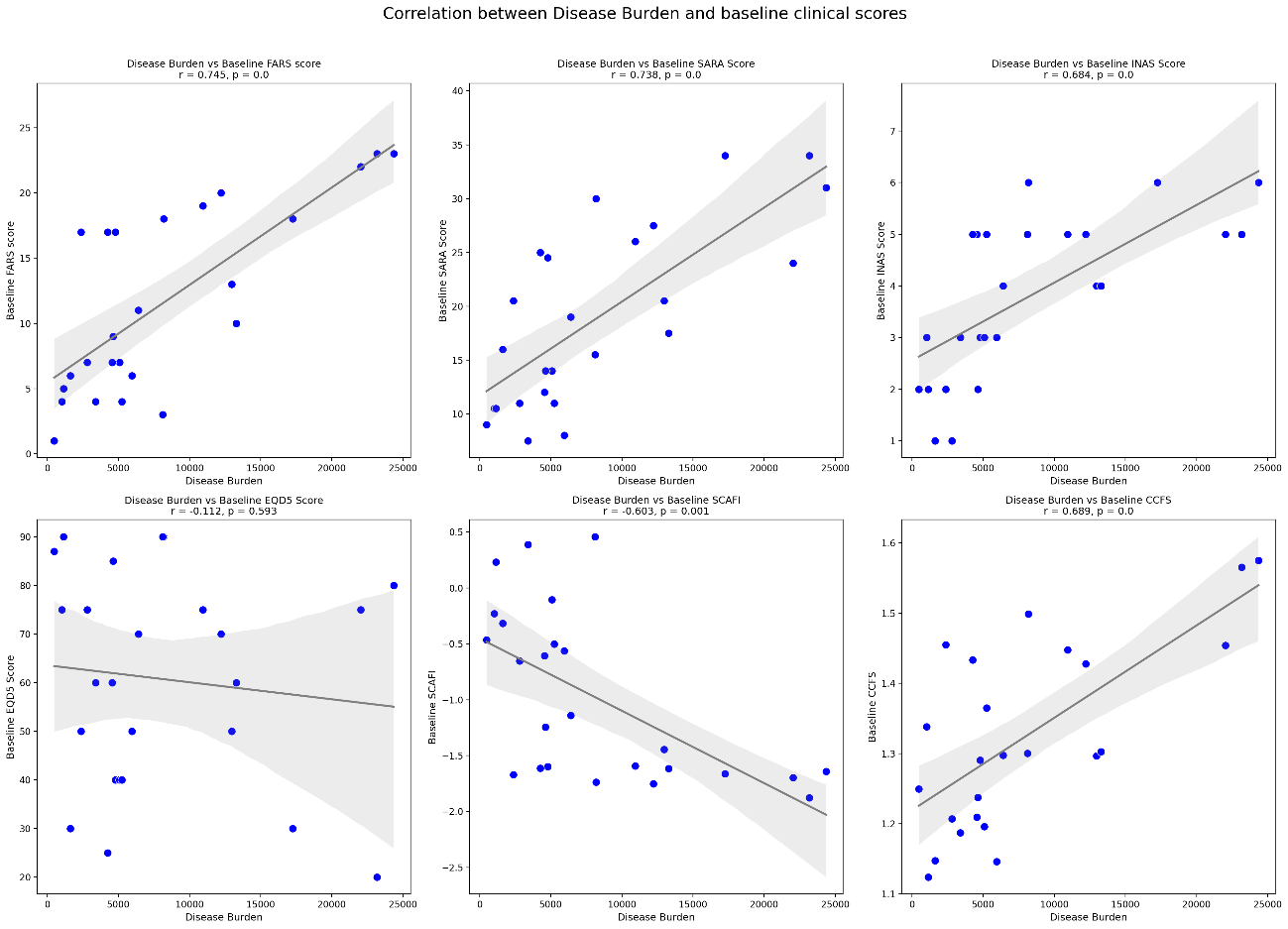

Supplement: Supplementary file 1 — Supplementary file1 Correlation between CSF NfL baseline levels (A), FXN baseline levels (B), GAA1 (C), age at onset (D) and Disease Burden (E) with scales (DOCX 611 KB) [file 415_2026_13812_MOESM1_ESM.docx]
